# Knowledge about Fertility in Croatia, Measured with the Croatian Version of the Cardiff Fertility Knowledge Scale (CFKS-Hr), in Relation to Attitudes toward Having a Child and Associated Factors in a Cross-Sectional Survey

**DOI:** 10.3390/nursrep14020063

**Published:** 2024-04-01

**Authors:** Ante Buljubašić, Deana Švaljug, Martina Mudnić Pulje, Ivana Gusar, Jelena Jerković, Iris Jerončić Tomić

**Affiliations:** 1 Department of Health Studies, University of Split, 21000 Split, Croatia; martinamudnicpulje7@gmail.com (M.M.P.); jjerkov@kbsplit.hr (J.J.); 2 Faculty of Health Studies Rijeka, University of Rijeka, 51000 Rijeka, Croatia; deana.svaljug@uniri.hr; 3 Department of Health Studies, University of Zadar, 23000 Zadar, Croatia; igusar@unizd.hr; 4 School of Medicine, University of Split, 21000 Split, Croatia; iris.jeroncic.tomic@mefst.hr

**Keywords:** fertility, fertility knowledge, reproductive decision-making, awareness

## Abstract

Background: Fertility is a crucial component of population maintenance and growth. A declining fertility trend has been observed over the past two decades, and it continues. The birth rate in the Republic of Croatia is continuously decreasing, which is insufficient to maintain its population. Objective: This research aimed to examine the level of fertility knowledge, factors influencing fertility knowledge, and the relationship between fertility knowledge and the decision to have a child. Methods: A cross-sectional study was conducted on a sample of mothers in five hospitals on the Adriatic coast of the Republic of Croatia, involving 1541 mothers, 18 years and older, from September 2021 to December 2023. The Cardiff Fertility Knowledge Scale (CFKS) was used for the research. The participants were divided into four groups according to age. The analysis was conducted using SPSS software (version 22.0). Descriptive statistics, chi-squared tests, *t*-tests, and Pearson’s correlations were used for the data analysis. Results: The average percent correct score on the CFKS was 51.8% (SD 22.6), with greater knowledge being significantly related to married status and higher education status (both *p* < 0.05). A total of 83% of mothers who have one child want to have more children. Fewer mothers who have two children want to have more children (42%), while only 11% of mothers who have three or more children want to have more children. There is no significant relationship between the CFKS and the importance of childbearing in the future (*p* = 0.12). Respondents indicated that they gained most of their knowledge from the internet (31%) and from the healthcare system (33%). Conclusions: The research results reveal a lack of fertility knowledge among participants, as well as an intention to have a child in the later stages of life. The lack of formal education on this topic leads to information gathering from friends, newspapers, television, and the internet. This study was not registered.

## 1. Introduction

The decline in fertility worldwide has been evident in the last two decades. Transition changes within families, prolonged education, and new lifestyles have resulted in a reduced number of children in families and delayed childbirth of the first child. The International Conference on Population and Development emphasizes fertility as one of the most important components of population growth [1,2]. The total world fertility rate has decreased from almost 5 births per woman in 1950–1955 to 2.5 in 2010–2015 [3]. Developed countries face the reality of childbirth at a later age and declining fertility [4,5]. In these countries, fertility is lower than 2.1 children per woman, which is necessary to maintain the population [6]. This approach to fertility can be explained by individuals focusing on their reproductive age, professional life, changes in lifestyle, and misinformation about fertility [7,8,9]. According to recent data, the birth rate in the Republic of Croatia (HR) is continuously decreasing (1.43 (2019), 1.42 (2020), 1.41 (2021), and 1.40 (2022)) [10]. This declining trend in fertility is not unexpected and was predicted by demographers [11]. Simultaneously, there has been an increase in the maternal age at the birth of the first child, which has risen from 26.5 (2005) to 29.7 (2021) in the European Union (EU) [12]. The lowest is in Bulgaria (26.5), and the highest is in Italy and Spain (31.6) [12]. In HR, the maternal age during that period increased from 26.5 (2005) to 29.2 (2021) [13]. Research on fertility knowledge has shown that it is insufficient in many countries [14,15,16,17]. A study conducted in Canada indicated the effectiveness of online education on fertility knowledge [18]. No research has been conducted in the Republic of Croatia that measures the level of knowledge about fertility and its related factors. Research conducted in Bosnia and Herzegovina showed a lack of knowledge about reproductive health that affects fertility [19]. Low fertility has numerous etiologies, and it is difficult to define specific factors. Research unequivocally shows that aging leads to a decline in fertility, occurring around the age of 32 and beyond [20]. Civilizational progress has led to social development, economic advancement, and, consequently, lifestyle changes [21]. A study conducted in 79 countries showed an average fertility knowledge test score of 56.9% [22]. Participants in Italy, Japan, Germany, Sweden, and other developed countries had a low level of fertility knowledge [23,24,25,26,27]. Interestingly, even healthcare professionals, doctors, nurses, and midwives, in some cases, showed a low level of fertility knowledge [28,29,30]. This is a consequence of the low level of fertility knowledge in the student population in conducted studies [17,31,32,33]. Research conducted in Japan has shown that a higher level of fertility knowledge leads to the decision to have a child earlier [34]. In HR, there are no recent studies assessing women’s knowledge of fertility. There is currently only research available that focuses on the correlation between a person’s level of education and their age at the time of their first child’s birth. A study conducted in 2022 analyzed data from 500 mothers and showed that women with a lower level of education give birth at an earlier age and had a higher number of births, while women with a higher level of education give birth at a later age and had a smaller number of births [35].

The aim of this research was to examine the level of fertility knowledge among mothers in the coastal area of Croatia, the associated factors influencing fertility knowledge, and the relationship between fertility knowledge and the decision to have a child. The obtained data enable a change in educational content and a more systematic approach to the topic of fertility.

## 2. Materials and Methods

### 2.1. Research Design 

A cross-sectional study was conducted on a convenience sample of mothers in five hospitals on the Adriatic coast of HR from September 2021 to December 2022. 

### 2.2. Participants and Materials 

The study included mothers who, during the research period, were in the maternity wards of five hospitals on the Adriatic coast of the Republic of Croatia. We approached the mothers on the second day after delivery. The researcher explained the purpose of the research to the mothers and asked them for their verbal consent to participate. If they gave verbal consent, the mothers were given a written informed consent to sign. The mothers were given a paper questionnaire in an open envelope. After filling out the questionnaire, the mothers put it in an envelope and closed it. No identification data were requested from the mothers. The inclusion criteria were 18 years of age or older, the absence of psychological problems determined by reviewing medical documentation, the birth of a child (APGAR score 8–10), and signing an informed consent confirming voluntary participation in the study. During the specified period, we collected data from 1573 participants. Through the analysis of completed questionnaires, we identified thirty-two incorrectly filled questionnaires, which were subsequently discarded. We analyzed data from 1541 participants.

### 2.3. Sample Size

The sample size was calculated based on the total population of mothers in the specified hospitals over a year, considering a 95% confidence level and a 2.5 confidence interval [36]. The required sample size for the study was 1332 participants.

### 2.4. Questionnaire

The questionnaire used in this study is the Cardiff Fertility Knowledge Scale (CFKS), developed by Boivin and colleagues (2013) [22]. CFKS measures the level of knowledge through 13 statements in three areas: indicators of reduced fertility, fertility misconceptions, and basic facts about infertility [35]. Statements are assessed as “correct”, “incorrect”, or “I don’t know”. A correct answer is scored one point, while an incorrect or “I don’t know” response is assigned 0 points. Results are presented as percentages of the maximum possible score. The CFKS has a Cronbach’s α coefficient of 0.82, a test–retest reliability of 0.82, and a consistency index of 0.95, measured in the observed student population. Approval for using CFKS was obtained from the authors. The questionnaire was translated into Croatian by two masters in English and tested on a group of 106 third-year students in the undergraduate studies of Nursing at the University Department of Health Studies, University of Split, to confirm its clarity. Pilot data were not included in the study.

### 2.5. Sociodemographic Data

Sociodemographic data are shown in Table 1. The questionnaire contains sociodemographic areas that are suitable for comparison with other research on the topic of knowledge about fertility. 

### 2.6. Childbirth Data

Childbirth data are shown in Table 2. Participants provided information on the number of children born, the age at the birth of the first child, and their intention to have more children in the future. In response to the question “Do you want to have more children in the future?” participants answered “yes” or “no.”

### 2.7. Data on Fertility Education

Two questions focused on fertility knowledge. The question “Have you been educated about fertility so far?” required a “yes” or “no” response. If the answer was “yes”, participants responded to the question “Where were you educated on fertility?” with options such as “family, friends, education system, internet, healthcare system, other”. The participants could choose more than one option regarding where they received their fertility education.

### 2.8. Data Analysis

Sociodemographic variables were presented using descriptive statistics. Categorical variables were compared using the chi-square test. *t*-tests and analysis of variance (ANOVA) were used to compare overall results among sociodemographic categories. Statistical significance was indicated by a *p*-value less than 0.05. The analysis was conducted using SPSS software (version 22.0) (IBM Corp., New York, NY, USA).

### 2.9. Ethical Principles

The research was conducted in line with the Helsinki Declaration. All procedures carried out in this research were approved by the Ethics Committees of all healthcare institutions where the research was conducted. Permission to use CFKS was obtained from the authors. Participants were informed about the research by the investigators and through informed consent, which was the first part of the survey questionnaire. By signing the informed consent, participants agreed to participate. Participation in the research was anonymous, voluntary, and confidential. To preserve anonymity, participants were asked not to provide any identification details or phone numbers on the survey questionnaire. All collected data are only accessible to the researchers.

## 3. Results

### 3.1. Sociodemographic Data

Table 1 displays the specifics of sociodemographic data. The participants were divided into four groups based on age status. It is important to highlight that the average age of the 169 mothers was over 30 years old.

Table 2 shows data on childbirth. 

### 3.2. Source of Knowledge on Fertility

Although participants show a positive attitude toward fertility education, they tend to avoid this topic due to a sense of discomfort, which is higher among participants outside of marital partnerships.

Moreover, 82% of participants provided positive answers to the question “Have you been educated about fertility?” (answered yes). They were then asked, “Where have you been educated about fertility?” The next question was “Do you think fertility education is necessary?” to which participants mostly responded positively (86%). When asked, “Do you feel uncomfortable discussing this topic in an environment where there are people who are not close to you?” participants mostly answered positively (75%), and this response was mainly given by individuals not in marital partnerships (72%). Media, i.e., television and newspapers, were not an important source (3%) according to Table 3.

Table 3 shows the results regarding sources of knowledge on fertility.

### 3.3. Knowledge of Fertility and Related Factors

Participants responded to fertility statements with an accuracy of 51.8% (SD 22.6). There were differences in responses between the observed groups regarding age; however, they were not statistically significant. The results obtained from the answers to the CFKS are shown in Table 4. 

Table 5 presents the factors associated with fertility knowledge. Factors that are statistically significantly related to CFKS are age, marital status, and education status. Factors that did not show statistical significance are age, economic status, and place of residence.

## 4. Discussion

### 4.1. Knowledge of Fertility

The research we conducted on a population of women who gave birth showed a low level of knowledge about fertility. There is a loss of fertility with aging in women. In the case of women, a slight decrease in women’s fertility has been estimated between the late 20s and the early 30s, followed by a more marked decrease from the mid to late 30s [20]. Similar results about fertility knowledge were obtained in other studies on fertility [22,27]. An international study showed that knowledge about fertility was at 59.7%, while the research we conducted showed that the level of knowledge about fertility was at 51.9%. Participants who had a high level of education in our study had better results [22]. In other studies, data clearly show that a higher level of fertility knowledge is associated with a higher level of education [18,22,23,34]. Results indicating a lack of basic facts about fertility available in the non-scientific literature are concerning. Mothers must develop the ability to take a critical approach and need to look for the necessary information regarding scientific literature analysis. Analysis of the obtained data shows that participants do not demonstrate sufficient knowledge about the effect of diseases on fertility while at the same time thinking that having healthy habits is sufficient for preserving fertility. The attitudes of participants at this level of fertility knowledge can lead to delaying the decision to become pregnant and downplaying the importance of risk factors for reduced fertility. The described results resemble the data from a conducted study wherein a lack of knowledge was shown to be the main factor in incorrect health behavior [22]. Participants with higher socioeconomic status showed a higher level of fertility knowledge, as confirmed in previous studies [22,27]. Although participants express a positive attitude towards fertility education, they avoid discussing this topic due to a feeling of discomfort. The feeling of discomfort is higher among participants who are not in a marital relationship. Similar data have been found in previously conducted studies [27,31]. Previous research has pointed out that individuals with higher levels of education have a higher level of health literacy [37,38,39,40]. The data obtained from this research emphasize the need for engagement of the education system (primarily secondary level and college level) to raise the level of health literacy and destigmatize the topic of fertility, with a special focus on participants who show a lower level of knowledge. Promotional activities need to be designed to empower the young population for critical thinking and making informed decisions related to fertility. Promotional activities must be designed and implemented by experts in the field of public health. Such activities have already shown their potential and contributed to changes in attitudes toward fertility, as confirmed by a study in Australia [41].

### 4.2. Decision on Childbirth

In this study, only 11% of participants stated that they did not want a child at all but that pregnancy occurred unplanned. Previous research on this topic has shown different data: Japan 18.0%, USA 14.2%, Sweden 4.1%, Hong Kong 19.1% [18,30,32,42]. Responses to the question “Do you want to have more children in the future?” showed that participants consider it important to have children during their lifetime. At the same time, results showed women have children at a later stage of life, which is in line with studies conducted in other areas [43]. Since we know that women decide on their first childbirth at an increasingly later age, these data are consistent with epidemiological data in Croatia [44]. The low level of fertility knowledge and the decision to have a child at a later age when fertility declines may be a consequence of economic and political changes in Croatian society. The factors of this trend are yet to be determined. The question is whether fertility education alone will bring a positive shift in Croatia, although research on this topic shows positive experiences [8].

Women’s fertility knowledge has contributed to a positive attitude toward the decision to have a child [30,32,43]. Nevertheless, despite this, the postponement of childbirth to a later age is an increasingly common decision for women [45,46]. This attitude is explained by the need for women to reconcile their maternal and professional roles [43]. Social changes have influenced women’s priorities, which are increasingly dedicated to education and participation in the labor market than their mothers and grandmothers were. This attitude leads to the decision to postpone childbirth [47,48].

Although most participants answered that they had prior knowledge of fertility, their knowledge was still poor. Most participants acquired knowledge about fertility through the influence of mass media such as the internet or television, and a smaller number within the education system. The results found in other studies are somewhat similar: Japan—internet and media 41.4%; USA—education system 46%, family 19%, media 35% [32,42]. Seeking information about fertility from insufficiently accurate and poor-quality sources is present not only in Croatian but also in other social communities. The fertility problem is recognized on a global scale. Due to these facts, WHO has emphasized the need to pay attention to reproductive health, which includes the field of fertility [49]. The recommendation for the health policies of individual countries is to establish an effective model of preventive activities aimed at promoting fertility. Some countries have recognized the fertility problem earlier, understood the importance of this issue, and defined a program to promote reproductive health and fertility to reduce the problem and its impact on demographic changes within society [50]. Education on fertility should be carried out in schools through existing health education topics and within the primary healthcare system. Such actions to raise the health literacy of the population have not yet been carried out in Croatia, although their success has been confirmed by research [51,52]. Of course, introducing this topic into the education system and the healthcare system, due to its specificity, requires additional education of educational staff [53]. It is also necessary to consider the possibility of controlling information on this topic in the media and the internet due to forming attitudes based on unreliable information. Individuals find these sources of information desirable due to the simplicity of access and finding answers to questions that interest them. Research has shown that the inclusion of the education system in a quality way contributes to an increase in knowledge about fertility [54,55,56]. The impact of greater knowledge on this topic is not to be overlooked in unfavorable situations, when a woman, despite knowledge and a positive attitude towards fertility, cannot become pregnant, which can result in feelings of anxiety [56,57]. The results of this study confirm the results from other studies, including that mothers think that having a healthy lifestyle annuls the effect of other risk factors.

Considering the results obtained in this research, in future activities, attention should be directed towards verifying the effectiveness of education interventions and defining risk factors in forming a positive attitude towards the fertility problem. The question remains whether increased knowledge translates to a change in behavior. Policies to increase fertility knowledge might also need to be accompanied by policies that reduce the social and economic costs of childbearing for women.

This relevant research also has its limitations. 

First, the study is a cross-sectional study with a convenience sample conducted on participants from one part of Croatia (coastal area) and does not reflect the entire population that might offer different responses. Second, the participants in this study are a vulnerable population because they delivered a baby and were in the maternity ward. We tried to reduce this deficiency by including mothers who gave birth to a healthy child and by excluding mothers who gave birth to a child at risk.

## 5. Conclusions

The research has revealed a lack of knowledge about fertility and a tendency to have the first child later in life when changes and a decrease in fertility potential are already occurring. It has been shown that the lack of formal education on fertility leads to gathering information from unreliable sources such as social media. There is a need to reorganize the education and healthcare systems at the primary healthcare level to provide adequate education on this topic because fertility decisions should be based on correct and complete information. On a broader societal level, programs need to be developed to promote reproductive health and fertility with a positive impact on individual quality of life and improvements in the overall demographic structure of society.

## Figures and Tables

**Table 1:** Sociodemographic characteristics of participants (N = 1541).

Age in Years		N (%)	*p*
Mean (SD)	30.8 (5.2)		
Range (categories)	18–24	177 (11)	0.356
	25–29	423 (28)	
	30–34	531 (34)	
	35<	410 (27)	
Range (total)	18–41		
**Marriage status**			
Married		1418 (92)	0.462
Single parent		123 (8)	
**Education status**			
Elementary school or less		260 (17)	0.364
High school		865 (56)	
College/university		416 (27)	
**Place of living**			
Urban		1278 (83)	0.034
Rural		263 (17)	
**Economic status**			
	551–1000 €	255 (17)	0.125
	1001–1500 €	938 (60)	
	1501–2000 €	229 (15)	
	>2001 €	119 (8)	

Data on childbirth.

**Table 2:** Data on childbirth.

	Categories	N (%)	*p*
**Age at the birth of the first child**			
Years	18–24	215 (14)	0.032
	25–29	596 (39)	
	30–34	513 (33)	
	35<	217 (14)	
**Number of children born**			
First childbirth		796 (52)	0.214
Second childbirth		493 (32)	
Third childbirth or more		252 (16)	
**Do you want to have more children in the future?**			
First childbirth			
	Yes	661 (83)	0.021
	No	135 (17)	
Second childbirth			
	Yes	207 (42)	
	No	286 (58)	
Third childbirth or more			
	Yes	28 (11)	
	No	224 (89)	

**Table 3:** Sources of knowledge on fertility.

Source of Knowledge	Categories	N, %	*p*
Education system	Total	123 (8)	
	18–24	11 (9)	0.453
	25–29	21 (17)	
	30–34	32 (26)	
	35<	59 (48)	
Family, friends	Total	345 (22)	
	18–24	73 (22)	0.287
	25–29	62 (18)	
	30–34	91 (26)	
	35<	119 (34)	
Television, newspapers	Total	46 (3)	
	18–24	2 (5)	0.614
	25–29	12 (26)	
	30–34	19 (41)	
	35<	13 (28)	
Healthcare system	Total	521 (33)	
	18–24	26 (6)	0.032
	25–29	85 (16)	
	30–34	168 (32)	
	35<	242 (46)	
Internet	Total	491 (31)	
	18–24	107 (21)	0.562
	25–29	175 (36)	
	30–34	122 (25)	
	35<	87 (18)	
Not sure	Total	46 (3)	
	18–24	18 (39)	0.425
	25–29	13 (28)	
	30–34	11 (24)	
	35<	4 (9)	

**Table 4:** Fertility knowledge.

Statements (T—True; F—False)	Total Correct Answer (%)	Categories	Correct Answer (%)
A woman is less fertile after the age of 36 years. (T)	63	18–24	(7)
		25–29	(41)
		30–34	(30)
		35<	(22)
A couple would be classified as infertile if they did not achieve a pregnancy after 1 year of regular sexual intercourse (without using contraception). (T)	41	18–24	(22)
		25–29	(28)
		30–34	(36)
		35<	(14)
Smoking decreases female fertility. (T)	62	18–24	(7)
		25–29	(18)
		30–34	(33)
		35<	(42)
Smoking decreases male fertility. (T)	61	18–24	(2)
		25–29	(23)
		30–34	(29)
		35<	(46)
About 1 in 10 couples are infertile. (T)	41	18–24	(24)
		25–29	(21)
		30–34	(34)
		35<	(21)
If a man produces sperm, he is fertile. (F)	62	18–24	(32)
		25–29	(31)
		30–34	(23)
		35<	(14)
These days, a woman in her 40s has a similar chance of getting pregnant as a woman in her 30s. (F)	71	18–24	(18)
		25–29	(12)
		30–34	(34)
		35<	(36)
Having a healthy lifestyle makes you fertile. (F)	26	18–24	(9)
		25–29	(18)
		30–34	(32)
		35<	(41)
If a man has had mumps after puberty, he is more likely to later have a fertility problem. (T)	67	18–24	(3)
		25–29	(17)
		30–34	(34)
		35<	(46)
A woman who never menstruates is still fertile. (F)	32	18–24	(17)
		25–29	(23)
		30–34	(37)
		35<	(23)
If a woman is overweight by more than 13 kg, then she may not be able to get pregnant. (T)	51	18–24	(21)
		25–29	(18)
		30–34	(29)
		35<	(32)
If a man can achieve an erection, it is an indication that he is fertile. (F)	61	18–24	(26)
		25–29	(21)
		30–34	(32)
		35<	(21)
People who have had a sexually transmitted disease are likely to have reduced fertility. (T)	35	18–24	(36)
		25–29	(28)
		30–34	(20)
		35<	(16)

**Table 5:** Factors related to fertility knowledge—univariate analysis.

	* CFKS Mean (SD)	T/F Value	*p*-Value
**Age**			
18–24	51.4 (21.3)	−1.42	0.17
25–29	53.2 (20.5)		
30–34	52.3 (23.1)		
35<	55.6 (24.7)		
**Marriage status**			
Married	56.5 (18.6)	3.22	0.01
Single parent	52.9 (22.5)		
**Education status**			
Elementary school or less	53.4 (19.7)	4.23	0.01
High school	56.8 (17.6)		
College/university	59.3 (24.7)		
**Economic status**			
551–1000 EUR	50.7 (21.3)	−1.34	0.35
1001–1500 EUR	53.4 (22.6)		
1501–2000 EUR	52.4 (19.7)		
>2001 EUR	52.8 (21.9)		
**Place of living**			
Urban	57.8 (15.8)	−1.29	0.26
Rural	51.6 (19.7)		
**Age at the birth of the first child**			
18–24	49.8 (24.6)	−1.25	0.23
25–29	52.3 (23.1)		
30–34	55.4 (18.6)		
35<	51.4 (22.4)		
**Do you want to have more children in the future?**			
One child	52.6 (22.4)	−1.32	0.12
Two children	54.1 (18.6)		
Three children	56.5 (18.2)		
Four children	53.3 (23.4)		

* The average percent correct score on the CFKS-C; T/F value: the value of *t*-tests and analysis of variance (ANOVA), * *p* < 0.05.

## Data Availability

The data used to support the findings of this study are available from the corresponding author upon request.

## Public Involvement Statement

No public involvement in any aspect of this research.

## Guidelines and Standards Statement

This manuscript was drafted against the STROBE criteria for reporting cross-sectional studies.
